# Reply: Prostate cancer, insulin and androgen deprivation therapy

**DOI:** 10.1038/sj.bjc.6601352

**Published:** 2003-10-28

**Authors:** S Lehrer

**Affiliations:** 1 Department of Radiation Oncology, Mount Sinai School of Medicine, New York, USA; 2 Veterans Affairs Medical Center, Bronx, New York, NY 10029, USA

**Sir**,

Stattin and Kaaks's data show that insulin levels increase with tumour stage, which is what we reported. They do not have a large enough sample size (sufficient statistical power) to conclude that there is no difference in the insulin levels of the groups in the table. I suggest that Stattin and Kaaks collect a larger sample after determining the sample size; they need to provide 90% power of detecting a significant (*P*=0.05 two-tailed) result. Also, they might want to measure fasting insulin before breakfast to reduce the variability of the data, since insulin levels fluctuate throughout the day and after meals. In the meantime, I refer Stattin, Kaaks and all interested readers to the article by Altman and Bland (1995), which eloquently explains the pitfalls of concluding clinical insignificance when differences between sets of small sample size are found to be statistically nonsignificant.
